# Genome-wide identification and molecular expression profile analysis of FHY3/FAR1 gene family in walnut (*Juglans sigillata* L.) development

**DOI:** 10.1186/s12864-023-09629-2

**Published:** 2023-11-08

**Authors:** Shengqun Chen, Yingfu Chen, Mei Liang, Shuang Qu, Lianwen Shen, Yajun Zeng, Na Hou

**Affiliations:** 1 Guizhou Academy of Forestry, Guiyang, 550005 Guizhou China; 2 Guizhou Province Forestry Science and Technology Extension Station, Guiyang, 550000 China; 3 Key Laboratory for Forest Resources Conservation and Utilization in the Southwest Mountains of China, Ministry of Education, Southwest Forestry University, Kunming, 650224 China; 4 Key Laboratory for Forest Genetics and Tree Improvement and Propagation in Universities of Yunnan Province, Southwest Forestry University, Kunming, 650224 China

**Keywords:** Walnut, FHY3, FAR1, Gene family, Kernel

## Abstract

**Background:**

*Juglans sigillata* L. (walnut) has a high economic value for nuts and wood and has been widely grown and eaten around the world. Light plays an important role in regulating the development of the walnut embryo and promoting nucleolus enlargement, which is one of the factors affecting the yield and quality of walnut. However, little is known about the effect of light on the growth and quality of walnuts. Studies have shown that far red prolonged hypocotyl 3 (FHY3) and far red damaged response (FAR1) play important roles in plant growth, light response, and resistance. Therefore, FHY3/FAR1 genes were identified in walnuts on a genome-wide basis during their growth and development to reveal the potential regulation mechanisms involved in walnut kernel growth and development.

**Results:**

In the present study, a total of 61 FHY3/FAR1 gene family members in walnuts have been identified, ranging in length from 117 aa to 895 aa. These gene family members have FHY3 or FAR1 conserved domains, which are unevenly distributed on the 15 chromosomes (Chr) of the walnut (except for the Chr16). All 61 FHY3/FAR1 genes were divided into five subclasses (I, II, III, IV, and V) by phylogenetic tree analysis. The results indicated that FHY3/FAR1 genes in the same subclasses with similar structures might be involved in regulating the growth and development of walnut. The gene expression profiles were analyzed in different walnut kernel varieties (Q, T, and F). The result showed that some FHY3/FAR1 genes might be involved in the regulation of walnut kernel ripening and seed coat color formation. Seven genes (*OF07056-RA*, *OF09665-RA*, *OF24282-RA*, *OF26012-RA*, *OF28029-RA*, *OF28030-RA*, and *OF08124-RA*) were predicted to be associated with flavonoid biosynthetic gene regulation cis-acting elements in promoter sequences. RT-PCR was used to verify the expression levels of candidate genes during the development and color change of walnut kernels. In addition, light responsiveness and MeJA responsiveness are important promoter regulatory elements in the FHY3/FAR1 gene family, which are potentially involved in the light response, growth, and development of walnut plants.

**Conclusion:**

The results of this study provide a valuable reference for supplementing the genomic sequencing results of walnut, and pave the way for further research on the FHY3/FAR1 gene function of walnut.

**Supplementary Information:**

The online version contains supplementary material available at 10.1186/s12864-023-09629-2.

## Background

The plants or crops are usually suffering from various biotic and abiotic stresses during their growth, which can severely affect their growth and yield, such as drought, light, temperature, salinity, waterlogging, mycosis, virus, and bacteria stress. Among them, light is the most important environmental factor for plant growth and development [1]. Therefore, in order to protect themselves from these adverse environmental restrictions on their growth and reproduction, plants have developed a series of typical complex coping mechanisms to trigger or repress certain gene expression for specific functions [2, 3]. Plants use three types of photoreceptors to perceive and monitor changes in the direction, duration, quality, and wavelength of light [4, 5]. Cryptochrome and phototrophotropin sense the blue / UV-A region of the spectrum, while photochromes primarily sense red (R) and far-red (FR) light wavelengths. In these photoreceptors, the phytochromes are most prominent and are encoded by five phytochromes (phyA to phyE) in Arabidopsis [6]. Studies have shown that FHY3 and FAR1 play essential roles in plant growth and development, which are two homologous proteins for the photo-induced phytochrome A (phyA)-controlled far-red responses and subsequent photoreaction in plants [6, 7]. Therefore, it has great value to identify the FHY3/FAR1 gene family and study its gene function for the growth and development of green plants or crops to improve the agronomic traits such as yield and quality.

The FHY3/FAR1 transcription factor family plays an important role in regulating plant growth, light response, and resistance. FHY3/FAR1 is derived from an ancient mutation-like transposase, which is a typical transcription factor that binds the FHY3/FAR1 binding site (FBS) cis-acting elements on the promoter in the form of homologous dimers or heterodimers to regulate downstream gene expression [6]. A molecular genetic study has identified two loci, FHY3 and FAR1, as two positive regulators specifically for phyA-mediated high-irradiance responses (HIRs) in response to FR wavelengths of light [6]. Previous research found that in Arabidopsis thaliana, functionally deficient mutants of fhy1, fhy2, and fhy3 show hypocotyl elongation in FR light but not in white light [8]. This result indicates their role in the light response of plants.

At present, the FHY3/FAR1 transcription factor family has been analyzed and reported in many plant species. In Arabidopsis, tea plants (*Camellia sinensis*), Eucalyptus (*Eucalyptus grandis* Hill ex Maiden), and peanuts (*Arachis hypogaea*) had 12, 25, 33, and 246 FHY3/FAR1 family members, respectively [6, 7, 9, 10]. However, there have been no studies on the FHY3/FAR1 gene or on the whole gene family analysis in walnut. In addition, genome-wide analyses of previous studies in Arabidopsis indicated that FHY3 has a number of putative targets, suggesting that FHY3 may have a wider range of functions in plant growth and development, while most of these gene functions remain unknown [11]. Therefore, further research on the gene function of FHY3/FAR1 members is also needed.

Walnut (2n = 32), as the oldest cultivated economic tree, is widely grown and eaten around the world due to its valuable nuts and wood, which originated in Central Asia and Southeast Europe [12]. *Juglans sigillata*, also called iron walnut, is the second most grown walnut tree species after *Juglans regia* (Persian walnut). According to the Food and Agriculture Organization of the United Nations (2017), China’s walnut production is 1.925 million tons, accounting for 47% of the world’s total walnut production. Walnuts have a high economic value in the food industry because of their rich nutrient content, such as fatty acids, polyphenols, flavonoids, steroids, carbohydrates, protein, dietary fiber, heart glycosides, melatonin, folic acid, and vitamins [13]. With the increasing walnut planting area, it has encountered various problems, including unstable yield and quality, and the plants are affected by various environmental factors that restrict the walnut’s normal growth and industry development [1]. Light plays a crucial determining role in regulating embryonic development and promoting kernel enlargement in walnut, which is one of the factors affecting the yield and quality of walnut. However, little information is obtained about the effects of light on the growth, development, and quality of walnuts. Recognition and study of the FHY3/FAR1 family members in walnut remain unknown.

In order to obtain sufficient genetic resources, revealing the growth and development of walnut and its response mechanisms to environmental stress such as light is extremely important. Therefore, it is necessary to identify the FHY3/FAR1 genes during the growth and development of walnut and to reveal the mechanism of growth and resistance regulation, which can provide insights for further research on the FHY3/FAR1 gene function of walnut response to light and quality breeding.

## Materials and methods

### Plant materials

Three walnut varieties were grown under the same environmental conditions and used for transcriptome sequencing in this study. These three walnut varieties have different colored pellicle, which includes the varieties of shajiwuren, hongguowuren, and songguowuren renamed F, Q, and T, respectively [14]. Wuren walnut, bred by sexual breeding in the Shangluo Walnut Research Institute and Shanyang County Forestry Station, comes from a local variety in Beigousi village, Shanyang County. The age of the three varieties of walnut are 45 years. Two-time point growth stages of the walnut fruit samples were collected at the July 1, 2020, fruit expansion stage (120 days post-anthesis, DPA) and September 16, 2020, maturity stage (harvest time, 165 DPA), respectively. Each group had three biological replicates. F1, Q1, and T1 represent 120 DPA, and F2, Q2, and T2 represent 165 DPA (Fig. 4). To avoid accidental errors, we mixed the samples from each group. Each sample was mixed with at least five kernels of the same walnut variety. After removing the green peel, wash the walnuts with clean water five times and collect kernel samples. All these walnut samples were obtained at the Institute for Forest Resources and Environment of Guizhou, Guizhou University, and quickly frozen in liquid nitrogen to store in a -80 refrigerator.

### FHY3/FAR1 gene identification in walnut

All known FHY3/FAR1 protein sequences in Arabidopsis were obtained from the TAIR (Arabidopsis Information Resource) database (http://www.arabidopsis.org/). Then, these Arabidopsis FHY3/FAR1 protein sequences were used for online tool BLASTP queries (E-value < 1e^− 5^) in the walnut whole genome to identify homologous FHY3/FAR1 protein sequences in walnut. The whole genome information of walnuts was obtained from the Research Institute of Forestry, Chinese Academy of Forestry [15]. The redundant proteins from the walnut genome were removed manually to obtain the real FHY3/FAR1 family members. The existence of the kinase domain structure and integrity is confirmed by the use of HMMER (https://www.ebi.ac.uk/Tools/hmmer/) corresponding to Pfam (http://pfam.xfam.org/) scanning to predict the protein walnut genome. In addition, with the fake members eliminated, these real walnut FHY3/FAR1 gene members were renamed in the present study as JsFAR1s. The gene transcript length and chromosome localization of the FHY3/FAR1 genes from walnut were calculated according to the method of Liu et al. (2023) [1]. The theoretical isoelectric point (PI) and molecular weight (Mw) of the all walnut FHY3/FAR1 proteins were calculated by using online tools on the ExPASy (https://web.expasy.org/compute_pi/) website.

### Chromosomal location and gene duplication analysis of FHY3/FAR1 members

The FHY3/FAR1 family members were mapped to chromosomal positions according to the location information of the walnut genome. Then, TBtools software was used to visualize the chromosomal location results [16]. The gene duplication events of FHY3/FAR1 members in walnut species were analyzed using MCScanX (Multiple Collinearity Scan Toolkit) software [17]. Multiple sequence alignments in FHY3/FAR1 members full-length amino acid sequences were analyzed by the ClustalW2 program [1, 18]. After identifying the duplicated FHY3/FAR1 genes, we calculated the Ka/Ks values, selection pressure, and selection mode.

### Gene structure and conserved domain analysis of FHY3/FAR1 members

The exon or intron structure information was obtained from the *Juglans sigillata* GFF file to understand the FHY3/FAR1 gene structure. Then, TB-tools software was used to visualize the gene structure of FHY3/FAR1 members [1, 16]. The conserved motif structure of FHY3/FAR1 protein members were analyzed using the online tool Multiple Em for Motif Elicitation (MEME, https://meme-suite.org/meme/tools/meme) in walnut. The NCBI (National Center for Biotechnology Information) Conservative Domain Database (https://www.ncbi.nlm.nih.gov/cdd/) was used to confirm the results [1].

### Phylogenetic analysis of FHY3/FAR1 members

Phylogenetic analysis was conducted on the newly identified 61 FHY3/FAR1 proteins of walnut and Arabidopsis. The FHY3/FAR1 protein sequences of Arabidopsis were obtained from the TAIR (https://www.arabidopsis.org/) database. MEGA (Molecular Evolutionary Genetics Analysis) version 7.0 software was used to perform multiple sequence alignments on the obtained FHY3/FAR1 protein sequences. Based on the results of multiple sequence alignments, the Neighbor-Joining algorithm is used to construct a phylogenetic tree, and the Replications of Bootstrap are set to 1,000 [1].

### Promoter-cis-acting element and secondary structure analysis

The 2 kb (non-coding region sequence) upstream region before the coding sequence (CDS) of members of the FHY3/FAR1 gene family was predicted [1]. Promoter-cis-acting elements are predicted and analyzed on the PlantCare website (http://bioinformatics.psb.ugent.be/webtools/plantcare/html/). Secondary structure analysis of FHY3/FAR1 proteins was conducted using the online prediction software of DETAIBIO (http://www.detaibio.com/tools/chou-fasman-forecast.html).

### RNA sequencing and gene expression profile analysis

The total RNA of walnuts was isolated from approximately 100 mg of frozen pericarp samples using the RNeasy plant mini kit (Qiagen) according to the manufacturer’s instructions. The RIN algorithm was determined by the Agilent Bio analyzer 2100 system (Agilent RNA 6000 Nano Kit, Agilent, Cat No. 5067 − 1511) for 18 cDNA library constructions (RNA integrity number > 7) on an Illumina HiSeq 2000 platform. After removing poly-N and low-quality reads, the clean reads were assembled, and the Q30 and GC contents were calculated by using Trinity [19]. Based on the results of our previous research, the FPKM (Fragments Per Kilobase of exon model per Million fragments mapped) value of each walnut gene was calculated, and the read counts of each gene were obtained by htseq-count for FHY3/FAR1 gene expression profile analysis [14, 19]. The differentially expressed genes were enriched in the Kyoto Encyclopedia of Genes and Genomes (KEGG) database to identify key metabolic pathways [20–22].

### Real-time quantitative RT-PCR detection

To verify the usability of the gene expression data and FHY3/FAR1 gene function for walnut growth, the relative transcript levels of eight candidate genes were determined using quantitative real-time reverse-transcription PCR (qRT-PCR). Gene-specific primer pairs were designed using Primer 4.0. Revert Aid First Strand cDNA Synthesis Kit (Fermentas, Lithuania) was used to reverse-transcribe total RNA in walnuts to obtain first-strand cDNA according to the manufacturer’s instructions. qRT-PCR was performed using the SYBR1 Green PCR Kit (Qiagen, 204,054). Plant actin was used as the internal reference to normalize the cDNA content. The mRNA expression level of genes was calculated using the 2^−ΔΔCt^ method [1, 19]. All genes were repeated three times.

### Statistical analysis of data

Data from three biological repeats were analyzed using GraphPad Prism 5, Excel 2013, and SPSS 20.0 and rendered as the means ± SD. A one-way Analysis of Variance (ANOVA) was performed, followed by Tukey’s significant difference test at *p* < 0.05. All data had three biological repeats. Differentially expressed genes were defined as genes with an FDR < 0.001 and a fold change > 2-fold [19]. A p-value < 0.05 was considered significant when identifying enriched GO terms, and a p-value < 0.05 was considered indicative of significantly enriched Kyoto Encyclopedia of Genes and Genomes (KEGG) pathways [20–22].

## Results

### Identification of FHY3/FAR1 family members in Walnut

In this study, amino acid sequences of the FHY3/FAR1 gene family in Arabidopsis thaliana and peanuts were used to BLAST (Basic Local Alignment Search Tool) the walnut genome. After deleting the fake gene members, a total of 61 FHY3/FAR1 gene family members of walnuts were obtained in this study (Table 1). We tentatively named the gene IDs of 61 FHY3/FAR1 family members in walnuts, namely JsFAR1-1 ~ JsFAR1-61. Except for 4 family members that were not mapped to chromosomes, the remaining 57 FHY3/FAR1 genes were mapped to each chromosome of walnut. The average length of amino acid sequences of FHY3/FAR1 in all walnuts was 297 aa, and the length distribution ranged from 117 aa to 895 aa (Table 1). The average isoelectric point (pI) of JsFHY3/FAR1 family members is 7.6, ranging from 4.2 to 9.8. The molecular weights (Mw) of each gene family member ranged from 13,329 to 102,402 (Table 1).

**Table 1:** Identification and information of FHY3/FAR1 gene family members in Walnut

Gene Namber	Gene ID	Chromosomal location	Amino acid length	Isoelectric point (pI)	Molecular weight (Mw)
JsFAR1-1	OF08124-RA	Chr 02	277	6.71	31236.04
JsFAR1-2	OF23566-RA	Chr 03	895	6.77	102402.35
JsFAR1-3	OF27118-RA	Chr 12	356	5.97	41261.21
JsFAR1-4	OF05621-RA	Chr 01	231	8.42	26960.88
JsFAR1-5	OF02131-RA	Chr 06	415	8.84	48483.48
JsFAR1-6	OF08125-RA	Chr 02	224	9.14	24792.87
JsFAR1-7	OF02230-RA	Chr 06	326	7.49	37702.59
JsFAR1-8	OF03129-RA	Chr 02	318	9.29	36443.5
JsFAR1-9	OF30211-RA	Chr 02	326	9.09	37854.8
JsFAR1-10	OF27337-RA	Chr 12	228	6.98	26706.57
JsFAR1-11	OF20968-RA	Chr 06	298	5.59	33004.84
JsFAR1-12	OF12739-RA	Chr 11	364	6.48	41603.2
JsFAR1-13	OF18387-RA	Chr 14	237	6.39	27814.99
JsFAR1-14	OF25919-RA	Chr 04	428	7.01	47333.95
JsFAR1-15	OF29114-RA	Chr 06	486	6.68	54717.47
JsFAR1-16	OF27402-RA	Chr 12	350	5.76	39960.02
JsFAR1-17	OF28030-RA	Chr 12	270	8.98	31054.39
JsFAR1-18	OF21567-RA	Chr 05	337	8.04	36649.86
JsFAR1-19	OF26570-RA	Chr 04	314	8.3	34310.61
JsFAR1-20	OF28029-RA	Chr 12	227	8.55	25724.43
JsFAR1-21	OF07764-RA	Chr 01	292	9.09	34021.42
JsFAR1-22	OF29079-RA	Chr 06	341	5.09	38829.38
JsFAR1-23	OF28028-RA	Chr 12	337	8.72	38840.53
JsFAR1-24	OF03008-RA	Un	249	5.79	28339.12
JsFAR1-25	OF21458-RA	Chr 05	275	6.7	31243.63
JsFAR1-26	OF29794-RA	Chr 02	250	4.93	27718.64
JsFAR1-27	OF20148-RA	Chr 01	374	9.28	41703.61
JsFAR1-28	OF26012-RA	Chr 04	286	6.61	31916.81
JsFAR1-29	OF28027-RA	Chr 12	256	5.85	30034.81
JsFAR1-30	OF28572-RA	Un	291	6.24	32844.79
JsFAR1-31	OF03007-RA	Un	255	6.03	29496.31
JsFAR1-32	OF04310-RA	Chr 11	366	9.75	41402.63
JsFAR1-33	OF03272-RA	Chr 15	334	9.38	38702.68
JsFAR1-34	OF07056-RA	Chr 01	255	9.22	29171.47
JsFAR1-35	OF20129-RA	Chr 01	246	7.57	27474.37
JsFAR1-36	OF09976-RA	Chr 13	268	6.96	29679.45
JsFAR1-37	OF22357-RA	Chr 03	314	9.13	35760.77
JsFAR1-38	OF26473-RA	Un	368	8.61	40416.59
JsFAR1-39	OF29653-RA	Chr 02	226	8.75	26065.64
JsFAR1-40	OF18917-RA	Chr 07	527	6.35	59610.19
JsFAR1-41	OF07221-RA	Chr 01	254	6.12	28827.19
JsFAR1-42	OF20368-RA	Chr 01	392	9.78	44983.63
JsFAR1-43	OF18333-RA	Chr 03	214	7.68	23902.09
JsFAR1-44	OF02323-RA	Chr 14	376	8.82	41525.97
JsFAR1-45	OF24282-RA	Chr 04	202	9.56	22865.66
JsFAR1-46	OF18064-RA	Chr 07	265	9.73	30687.74
JsFAR1-47	OF02443-RA	Chr 14	282	5.68	31833.45
JsFAR1-48	OF27373-RA	Chr 12	197	9.36	22191.45
JsFAR1-49	OF29019-RA	Chr 08	120	4.2	13329.61
JsFAR1-50	OF13566-RA	Chr 11	150	6.21	16980.28
JsFAR1-51	OF11658-RA	Chr 10	180	5.76	20220.54
JsFAR1-52	OF11410-RA	Chr 10	300	5.35	33448.51
JsFAR1-53	OF23926-RA	Chr 04	338	8.65	38533.31
JsFAR1-54	OF27447-RA	Chr 12	321	8.46	35875.96
JsFAR1-55	OF14355-RA	Chr 09	272	8.8	30257.58
JsFAR1-56	OF17018-RA	Chr 05	237	5.49	25838.76
JsFAR1-57	OF02328-RA	Chr 14	117	5.53	13552.21
JsFAR1-58	OF09665-RA	Chr 13	203	9.59	23893.39
JsFAR1-59	OF29668-RA	Chr 02	201	8.75	22656.69
JsFAR1-60	OF11682-RA	Chr 10	271	9.25	30460.64
JsFAR1-61	OF04308-RA	Chr 11	203	9.69	22956.82

### Chromosome localization of FHY3/FAR1 members in Walnut

**Fig. 1:**
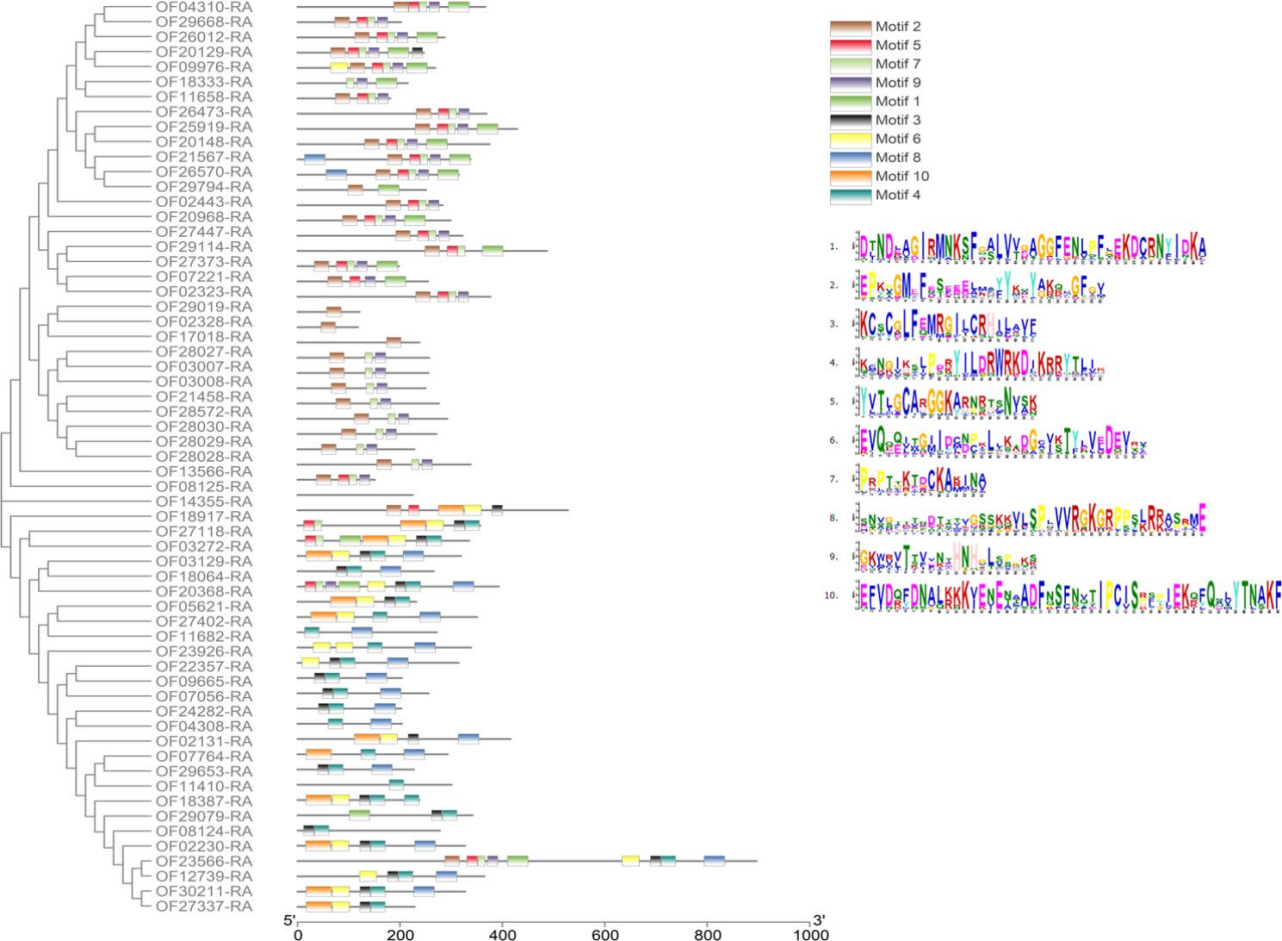
Location and distribution of FHY3/FAR1 gene family members in walnut chromosomes. The ruler on the left indicates the length (Mb) of the walnut chromosome. The yellow letters (Chr01 to Chr15) indicate the chromosome number of the walnut. The font marked in red indicates the ID number of the FHY3/FAR1 gene family in the walnut genome

In this study, the location information of each FHY3/FAR1 gene family member was used to map each FHY3/FAR1 gene to the chromosome of walnut. The results showed that the 61 gene family members were unevenly distributed on 15 chromosomes of walnut, but there was no FHY3/FAR1 gene on chromosome 16 (Fig. 1). There were 9 FHY3/FAR1 gene members on chromosome 12, followed by 7 genes on chromosomes 01 and 02. There were also four genes on Scaffold 21, 145, 358, and 538. Some tandem repeats of the FHY3/FAR1 genes were found on chromosomes 1 to 15 in walnut. For example, OF08124-RA and OF08125-RA are located on chromosome 02; OF04308-RA and OF04310-RA are located on chromosome 11; OF28027-RA, OF28028-RA, OF28029-RA, and OF28030-RA are located on chromosome 12; OF02323-RA and OF02328-RA are located on chromosome 14 (Fig. 1).

### Gene structure and conserved domain analysis of FHY3/FAR1 genes in Walnut

In this study, the gene structure of the FHY3/FAR1 family members of Walnut was analyzed. The results showed that all 61 family members had complete gene structure, containing at least one exon and intron (Figure S1). The conserved domain analysis of 61 family members showed that all family members contained either the FHY3 or FAR1 domains (Figure S2). Of these genes, 19 contain only the FAR1 domain, and 39 contain only the FHY3 domain. JsFAR1-40 (OF18917-RA) and JsFAR1-33 (OF03272-RA) have two FHY3 domains encoding 527 and 334 amino acids, respectively (Figure S2). OF29019-RA and OF17018-RA both contain a complete FHY3 domain and a partial FAR1 domain. These results indicated that the identification of 61 FHY3/FAR1 gene family members in walnut was accurate.

**Fig. 2:**
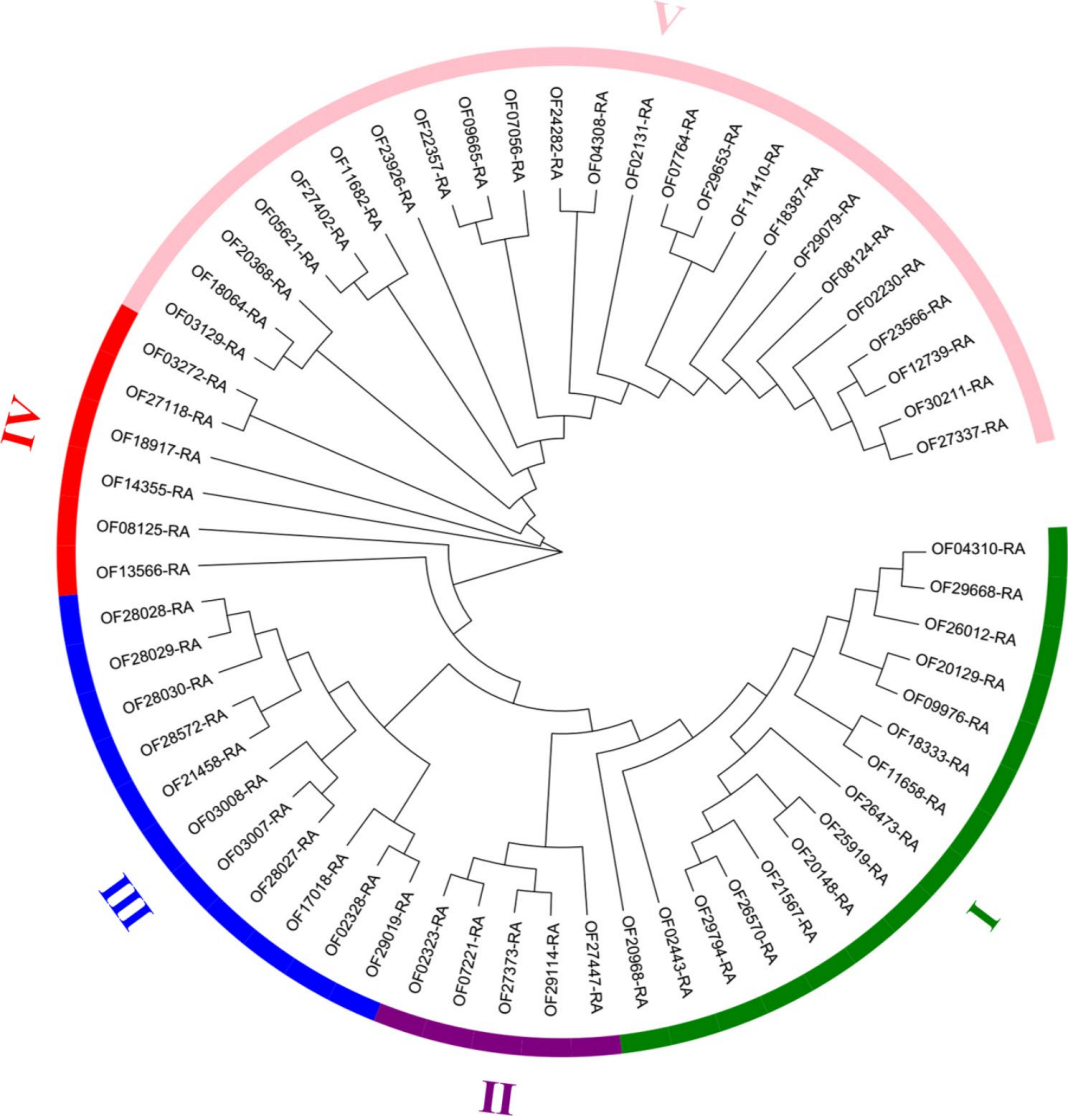
Motif-domain analysis of FHY3/FAR1 family members in Walnut. The evolutionary tree of the FHY3/FAR1 gene family is shown on the left. On the right side of the figure are the schematic diagram of the Motif’s color blocks and the sequence of 10 Motifs of FHY3/FAR1 family members

Therefore, motif prediction and analysis were performed on these walnut gene family members. The result indicated that all 61 FHY3/FAR1 genes contained at least one motif domain, and the FHY3/FAR1 gene member domains were more similar with similar gene clusters (Fig. 2). This suggests that these genes may have similar functions in plant growth and development regulation. There were 33 members containing motif 2, 24 members containing motif 6, 20 genes containing motif 10, and 18 genes containing motif 1. Among them, OF20919-RA, OF02328-RA, and OF17018-RA were clustered into one cluster, and only motif 2 was contained in them (Fig. 2). Other members of the walnut FHY3/FAR1 gene family all contained more than three motifs, indicating functional differences among these members.

### Analysis of evolutionary relationships between FHY3/FAR1 family members in Walnut

**Fig. 3:**
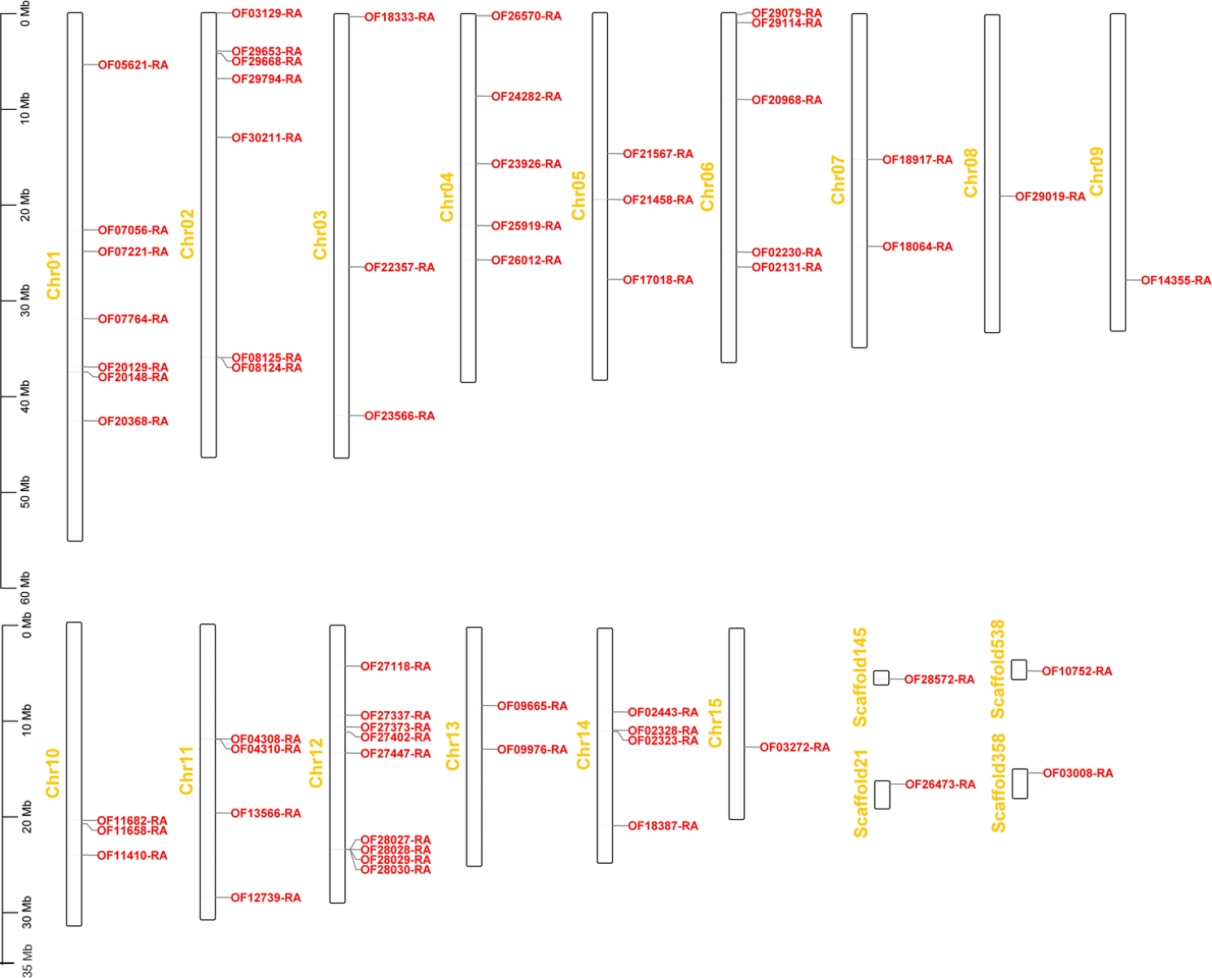
Phylogenetic tree and cluster analysis of FHY3/FAR1 family members in Walnut. Different line colors and Roman numerals (I, II, III, IV, and V) in the figure indicate subcategories of FHY3/FAR1 family members

To explore the evolutionary relationship between FHY3/FAR1 gene family members in Walnut. In this experiment, a phylogenetic tree was constructed for these 61 gene members (Fig. 3). We divided these genes into five subclasses based on their clustering results. Subclass I contained 15 gene members, subclass II contained 5 gene members, subclass III contained 11 gene members, subclass IV contained 6 gene members, and subclass V contained 24 gene members (Fig. 3). The closer the family members were in phylogenetic relationship, the more similar the gene structure and domain, indicating that FHY3/FAR1 genes of different subclasses may have different functions, and genes with similar structure might be involved in regulating the growth and development of walnut (Fig. 3).

### FHY3/FAR1 genes involved in walnut kernel development and seed coat color change

**Fig. 4:**
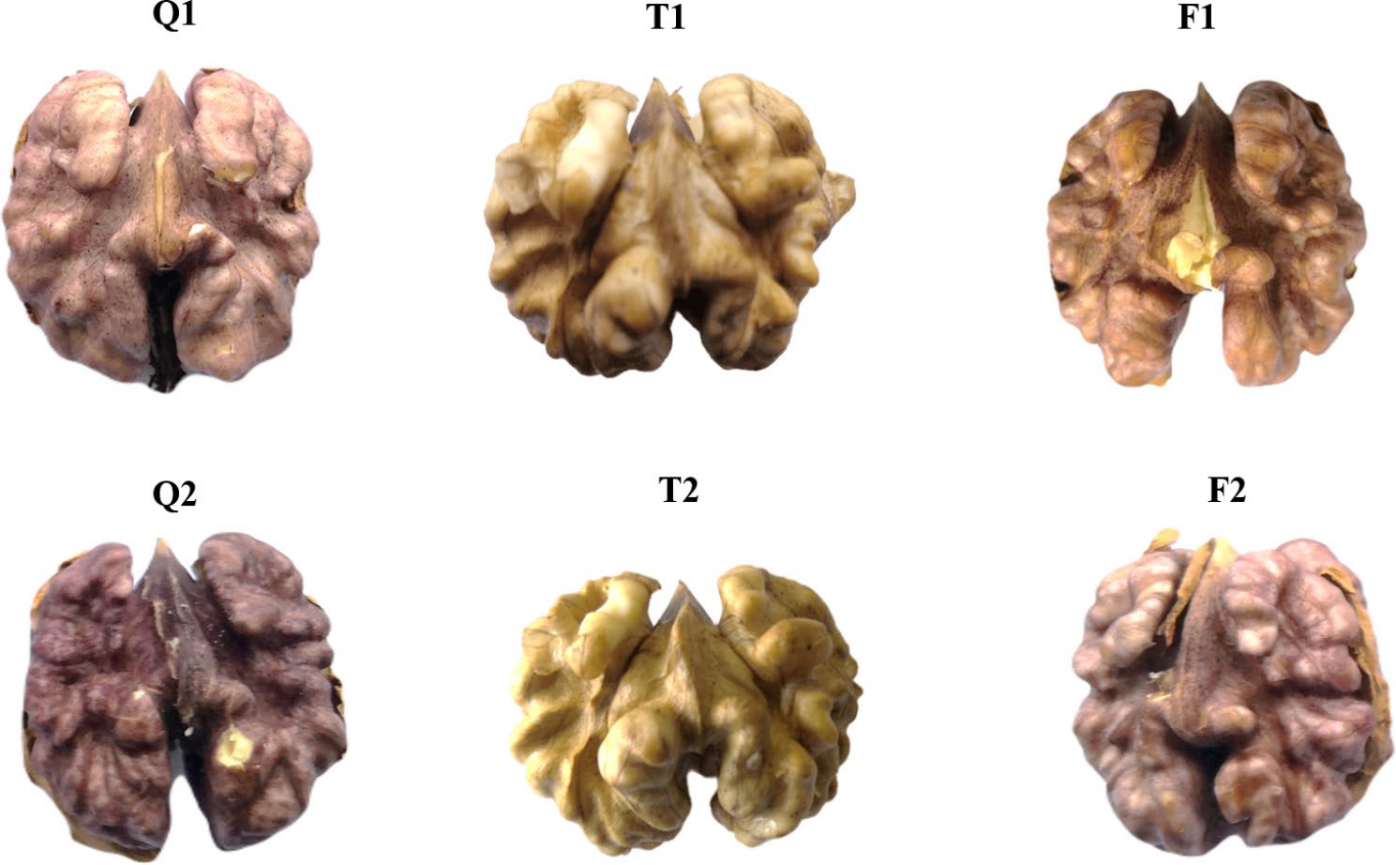
Phenotypes of Hongguo Wuren (Q), Songhe Wuren (T), and Sachijiwuren (F) in walnut kernels at different developmental stages. F1, Q1, and T1 represent 120 days post-anthesis (DPA), and F2, Q2, and T2 represent 165 DPA

In order to verify the hypothesis of FHY3/FAR1 gene function in walnut from previous results, three different walnut varieties, Hongguo Wuren (Q), Songhe Wuren (T), and Sachijiwuren (F), were selected for transcriptome sequencing at two stages of kernel development (immature and mature stages). The expression profile and regulation mechanism of the FHY3/FAR1 gene in walnut during maturation were analyzed. Q walnut seed coat is red after maturity, T walnut seed coat is yellow after maturity, and F walnut seed coat is pink after maturity (Fig. 4).

### RNA-Seq profile analysis of FHY3/FAR1 family members in Walnut

**Fig. 5:**
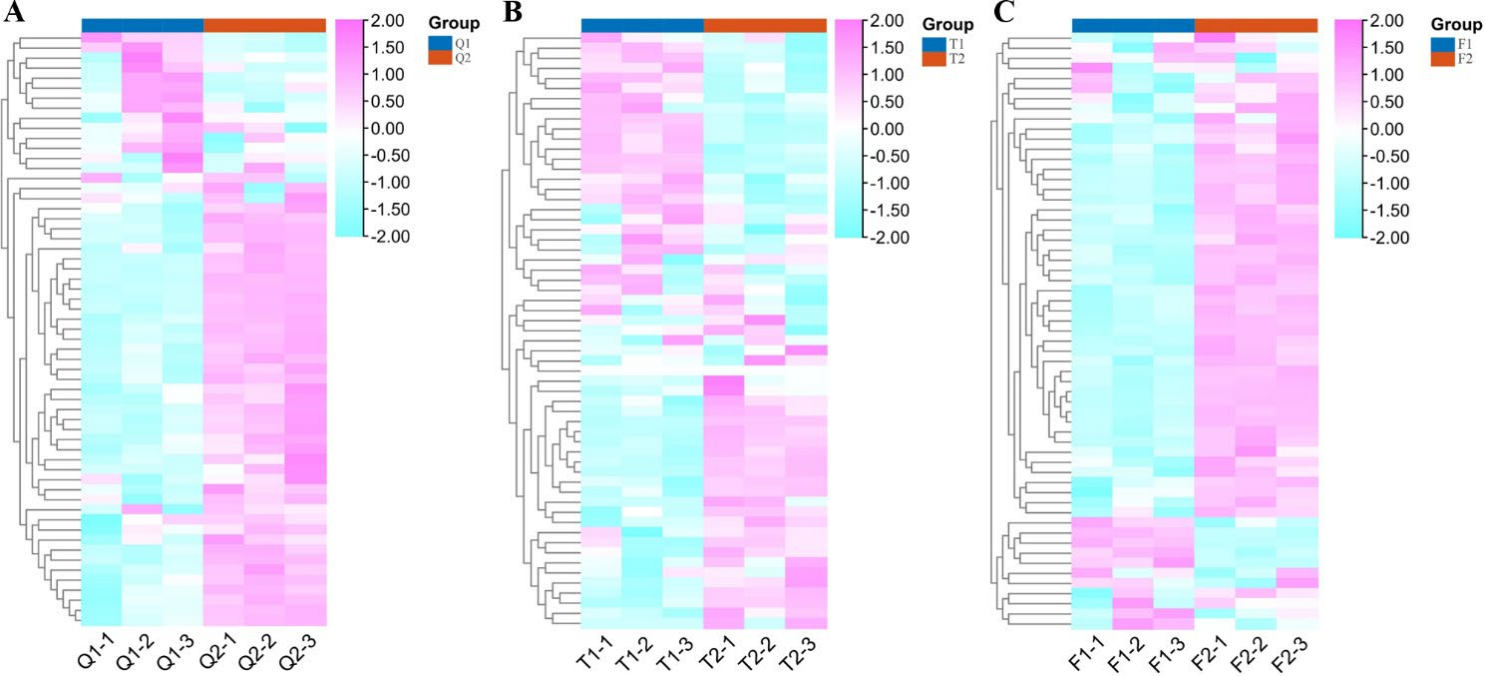
Expression profiles of the FHY3/FAR1 gene family in Q, T, and F walnut kernels at different developmental stages. The color bar on the right, purple, indicates a significant increase in gene expression level. A sky-blue color indicates a low gene expression level. *p* < 0.05

In the Q variety, most of the FHY3/FAR1 gene family members in walnuts showed up-regulated expression in Q2 (maturity stage) compared with Q1 (Fig. 5A). In T, about half of the FHY3/FAR1 gene family members were up-regulated and the other half were down-regulated at the T2 stage (Fig. 5B). In variety F, compared with F1, most members of the FHY3/FAR1 gene were up-regulated in the F2 stage (Fig. 5C). Among them, JsFAR1-10 (OF27337-RA), JsFAR1-13 (OF18387-RA), and JsFAR1-21 (OF07764-RA) were significantly up-regulated during the maturation of Q, T, and F varieties. JsFAR1-2 (OF23566-RA), JsFAR1-7 (OF02230-RA), and JsFAR1-3 (OF27118-RA) were significantly up-regulated during the maturation of Q and F varieties, while significantly down-regulated during the maturation of T varieties. However, JsFAR1-1 (OF08124-RA) was significantly down-regulated in all three walnut varieties after maturity. In addition, we also found that the expression level of FHY3/FAR1 genes in walnut at different developmental stages was correlated with the seed coat color formation of walnut. For example, the expression of FAR1 genes were up-regulated during the maturation of Q and F varieties, which resulted in a darker seed coat color (Figs. 4 and 5). However, the seed coat color of variety T changed little before and after maturity, and the expression of FHY3/FAR1 family members did not show an obvious tendency.

### Prompter analysis of FHY3/FAR1 genes in walnut

The potential cis-acting elements were predicted in the 2 kb sequence (non-coding region sequence) before the CDS sequence (start codon) of the FHY3/FAR1 gene family. A total of 19 main cis-acting elements were distributed in the 2 kb promoter regions of FHY3/FAR1 gene family members, including abscisic acid responsiveness (142), gibberellin responsiveness (59), light responsiveness (608), methyl jasmonate (MeJA) responsiveness (164), low-temperature responsiveness (40), flavonoid biosynthetic genes regulation (7), salicylic acid responsiveness (18), seed-specific regulation (6), auxin responsiveness (62), defense and stress responsiveness (26), zein metabolism regulation (47), drought-inducibility (41), 60 K protein binding site, endosperm expression (16), anaerobic induction (151), cell cycle regulation (4), circadian control (9), wound-responsive element (3), and meristem expression (27) (Additional file 1). Among them, light responsiveness is the most cis-acting component, followed by MeJA responsiveness, anaerobic induction, and abscisic acid responsiveness. Among them, seven FHY3/FAR1 gene family members possess flavonoid biosynthetic gene regulation cis-acting elements. This might be related to the seed coat color formation during the ripening process of Q, T, and F walnut varieties, which include OF07056-RA, OF09665-RA, OF24282-RA, OF26012-RA, OF28029-RA, OF28030-RA, and OF08124-RA.

### RT-PCR verification

**Fig. 6:**
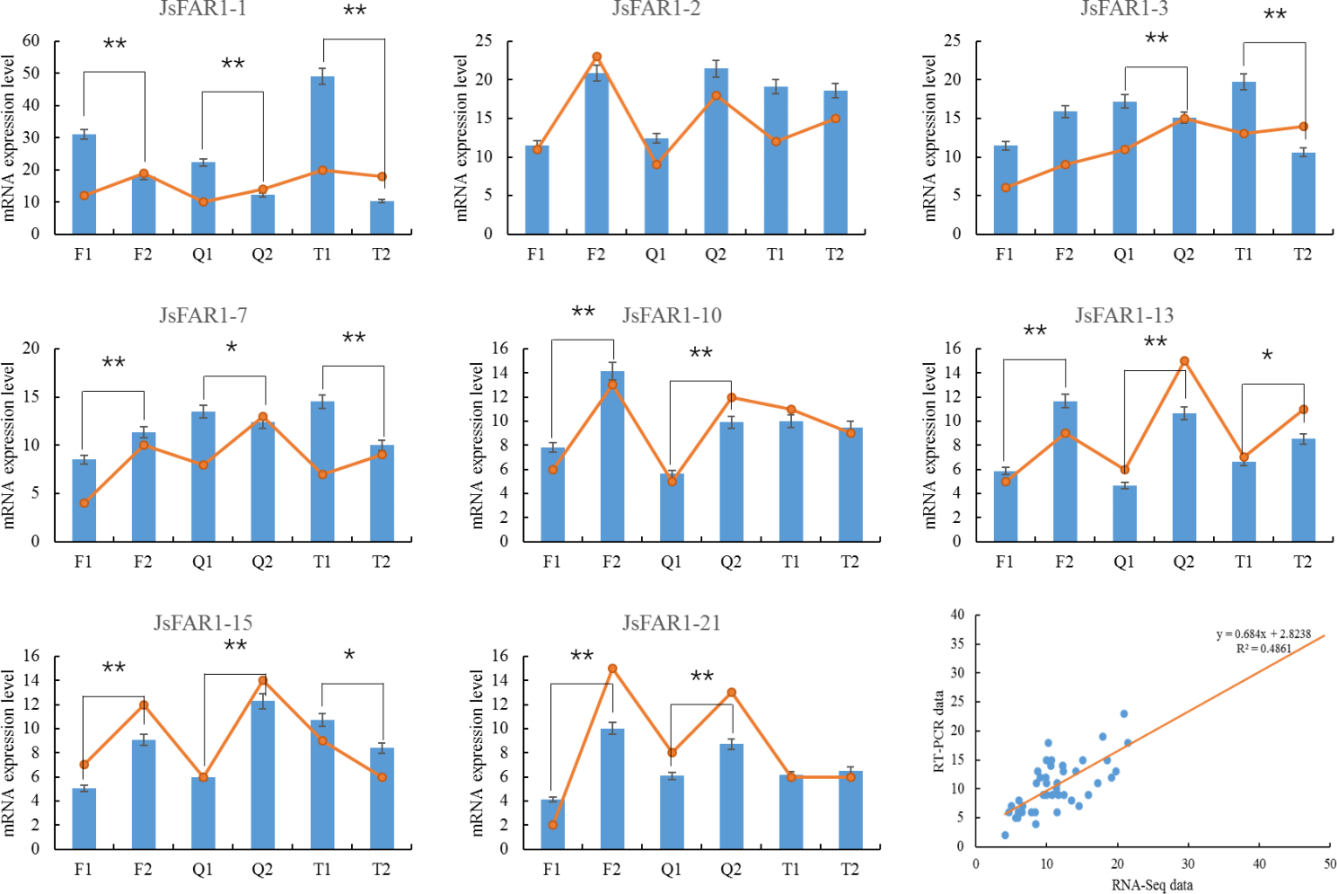
RT-PCR verification of candidate genes of the FHY3/FAR1 gene family in walnuts. The uppercase letters in the horizontal coordinates are “Hongguo Wuren (Q), Songhe Wuren (T), and Sachijiwuren (F)”. The numbers (1 and 2) next to the letters in the horizontal coordinate represent two developmental stages (sampling periods) of different walnut varieties. The column in the figure represents the gene expression level detected by RT-PCR, and the broken line in the column figure represents the gene expression level (FPKM value) of the RNA-Seq profile. The scatterplot in the lower right corner showed the correlation analysis between RT-PCR and RNA-Seq results (*p* < 0.01). * in the bar chart indicates the significance of the difference comparison (*, *p* < 0.05; **, *p* < 0.01)

In order to verify the accuracy and reliability of the RNA-Seq detection results in this experiment. The previously screened candidate FHY3/FAR1 genes were detected by RT-PCR. These candidate genes include JsFAR1-1, JsFAR1-2, JsFAR1-3, JsFAR1-7, JsFAR1-10, JsFAR1-13, JsFAR1-15, and JsFAR1-21. The results showed that the expression levels and trends of these genes in the RNA-Seq profile were consistent with those in the RT-PCR detection results (*p* < 0.05) (Fig. 6). A correlation analysis was conducted between RT-PCR detection results and RNA-Seq data, and the results confirmed the reliability and authenticity of the data results analyzed in this experiment (*p* < 0.01).

## Discussion

Walnuts are among the oldest foods known today, and they are grown throughout temperate zones of the world for nut production. Fresh walnuts are delicious, crispy, and more nutrient-denser than dried walnut products [23]; therefore, they are an ideal alternative. Due to the rapid growth of walnut consumption, there is a great demand for walnut products [24]. It is of great economic and productive value to study the development and quality formation mechanisms of walnut kernels. Previous studies have shown that transcription factors are important regulatory factors for gene expression and play a key role in signal switching networks during plant development. They directly up-regulate or inhibit the expression of target genes, thus interrupting the interaction between different genes that could potentially be involved in signaling pathways [25]. In this study, three walnut cultivars with different quality and kernel color were used for detailed analysis and study of the FHY3/FAR1 transcription factor family on a genome-wide level at different developmental stages.

Several previous studies suggested that FHY3/FAR1 have multiple functions in different physiological and developmental processes of plants, such as abiotic stress responses (salt and temperature), flowering, reactive oxygen species (ROS) homeostasis, chlorophyll biosynthesis, UV-B signaling, circadian clock entrainment, and abscisic acid (ABA) signaling [7]. However, the FHY3/FAR1 gene family has not been reported in walnut, and the specific biochemical functions of FHY3 and FAR1 in other biological processes other than phytochrome signaling remain unclear and need to be further clarified and elucidated [26]. Therefore, we performed genome-wide identification of the FAR1/FHY3 gene family in walnut by using the gene expression profile and bioinformatics analysis. In this experiment, 61 FAR1 gene family members were successfully identified on 15 chromosomes of walnut (excluding chromosome 16). This may be related to the deletion or copy loss of the walnut FHY3/FAR1 family during evolution.

Previous studies have reported that sequences of FHY3/FAR1 gene family members are homologous and conserved among land plants, which are considered the founding members of the FRS and FRS-RELATED FACTOR gene families, and the proteins encoded by these genes might be involved in different signaling processes in response to various biological and abiotic stresses [6, 26–28]. In this study, the conserved domains of the identified walnut gene families were analyzed, and it was found that these genes only contained the FHY3 and FAR1 domains, and had similar gene structures. In addition, FRS-like proteins have been found in other plant species, including monocotyledonous plants, suggesting that the protein family was conserved during the evolution of the plant kingdom [29]. Therefore, they could potentially play a unique and important role in plant growth and development.

Due to morphological limitations, plants often use their complex sensory systems to monitor their environment and adapt to growth. As a result, plants develop a range of defense and perception systems, such as light. Light plays an essential role in the growth and development of plants throughout their lives for seed germination, seedling and stem elongation, phototropism, chloroplast movement, circadian rhythms, and flowering [2, 5–7, 9, 26, 27]. In this study, the upstream 2 kb promoter sequences of 61 FHY3/FAR1 gene families were identified by cis-acting element analysis. The present results showed that light responsiveness, methyl jasmonate (MeJA) responsiveness, and abscisic acid responsiveness had the largest number of elements. The present results agree with the previous research reports that FHY3 and FAR1 might directly regulate the expression of acute photoreactive ELF4 in the subjective night period [28]. In addition, studies have shown that the light signaling factors FHY3 and FAR1 regulate plant immunity by regulating chlorophyll biosynthesis [29]. Therefore, combined with previous research reports and the results of this experiment, we speculated that some members of the FHY3/FAR1 gene family in walnut might also have the functions of light response and immune response.

In recent years, studies have shown that FHY3/FAR1 appears to constitute a key transcriptional signaling factor that coordinates the expression of downstream genes to ensure optimal plant growth, development, and immunity to various internal and external signals. In Arabidopsis thaliana and rice plants, the FHY3/FAR1 gene controls the accumulation of photosensitive pigments through FHY1 and is associated with the photosensitive pigment signaling pathway [26, 30]. Studies have shown that FHY3 and FAR1 play a critical role in mature plants responses to dark light conversion, and their functional loss shows an exaggerated shade avoidance phenotype in *Arabidopsis Thaliana* [30–32]. Similarly, the FHY3/FAR1 genes might also play an important regulatory role in the light response of walnut. Five phyA-phyE genes have been reported in *Arabidopsis Thaliana*, encoding different phytochromes that sense red (R) and FR light and regulate various growth and development processes in plants, including seed germination, stomatal membrane opening, chlorophyll synthesis, low-quality growth, and flower initiation [4, 33]. The regulatory functions of phyB and phyE gene products under continuous R light and white light are similar, and PHYB plays a dominant role. These gene family members identified in this study could also play an important role in regulating the development and quality formation of walnut kernels.

The phyA is primarily responsible for very low flux responses and FR light dependent high irradiance responses, including inhibition of hypocotyl elongation, opening of the apical hook, cotyledon expansion, anthocyanin accumulation, and green blocking of FR preconditioning [26]. In this study, we found flavonoid biosynthetic gene regulation function in the promoter cis-acting elements of seven FHY3/FAR1 gene family members in walnuts. Flavonoid synthesizing elements are potentially related to anthocyanin accumulation, which potentially leads to the color formation of walnut seed coats. In addition, cell cycle regulation elements were identified in four FHY3/FAR1 gene members. FAR1 appears to act downstream of the regulation cascade as a link between the signal transduction pathway and the cell cycle in yeast [34]. Twenty-six FHY3/FAR1 genes had cis-acting elements for defense and stress responsiveness. Loss-of-function of FHY3 and FAR1 resulted in increased ROS accumulation and sensitivity to oxidative stress in various plant species [35]. These results suggested that the 61 FHY3/FAR1 gene family members identified in this study could potentially play an essential role in responding to light, regulating the development of walnut kernels, and participating in the color change of the seed coat.

### Electronic supplementary material

Below is the link to the electronic supplementary material.

Supplementary Material 1

Supplementary Material 2

## Data Availability

The datasets generated and/or analysed during the current study are available in the [NCBI] repository, accession number [PRJNA965957].
