# Supplementary material for: Genome-wide identification and molecular expression profile analysis of FHY3/FAR1 gene family in walnut (Juglans sigillata L.) development
Source: BMC Genomics. 2023 Nov 8;24:673. doi: 10.1186/s12864-023-09629-2 (PMC10634098; doi:10.1186/s12864-023-09629-2)
Supplement: Supplementary file 2 — Supplementary Material 2 [file 12864_2023_9629_MOESM2_ESM.docx]

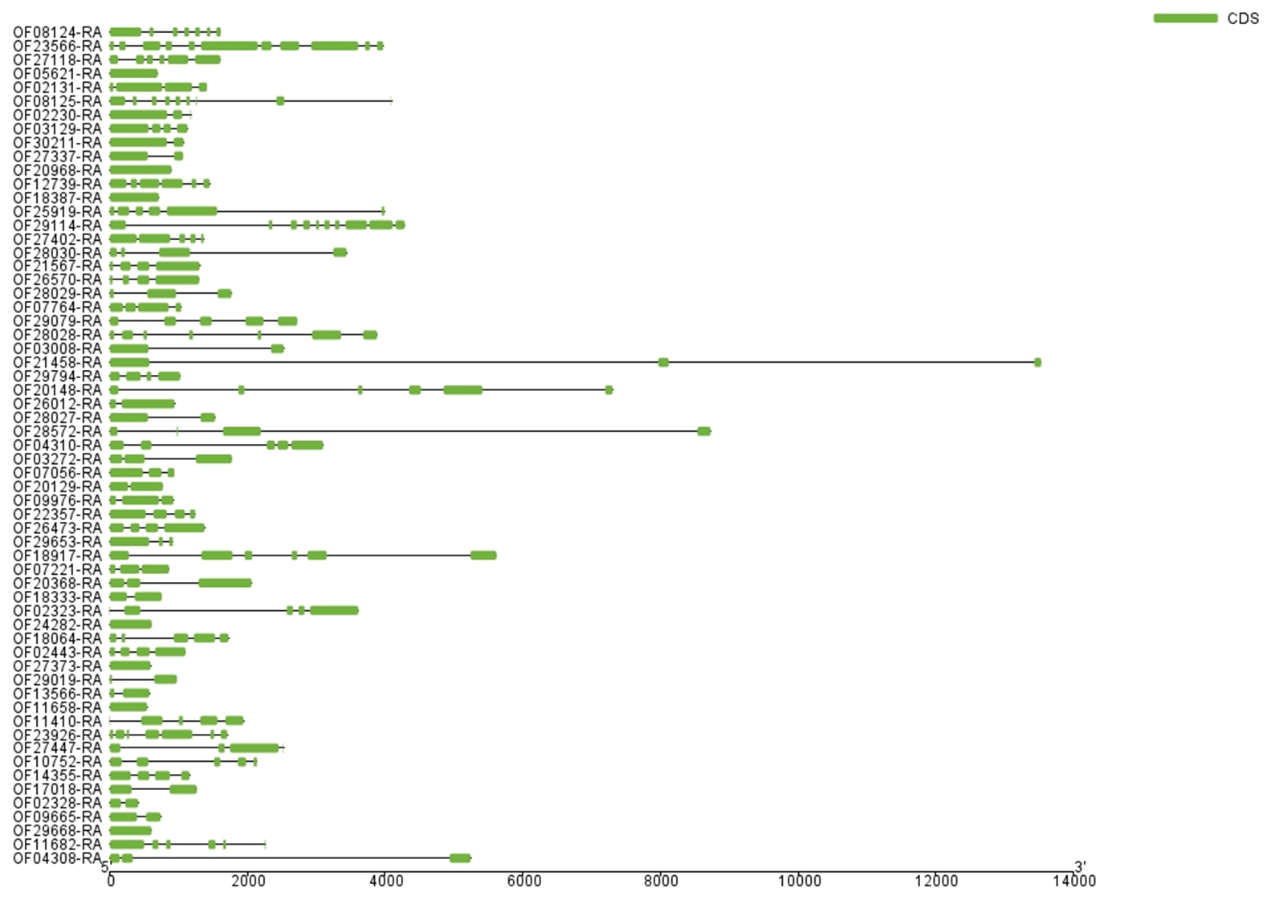


Figures S1. Gene structure analysis of FHY3/FAR1 gene family in Walnut. Solid black lines represent introns and green bars represent CDS.


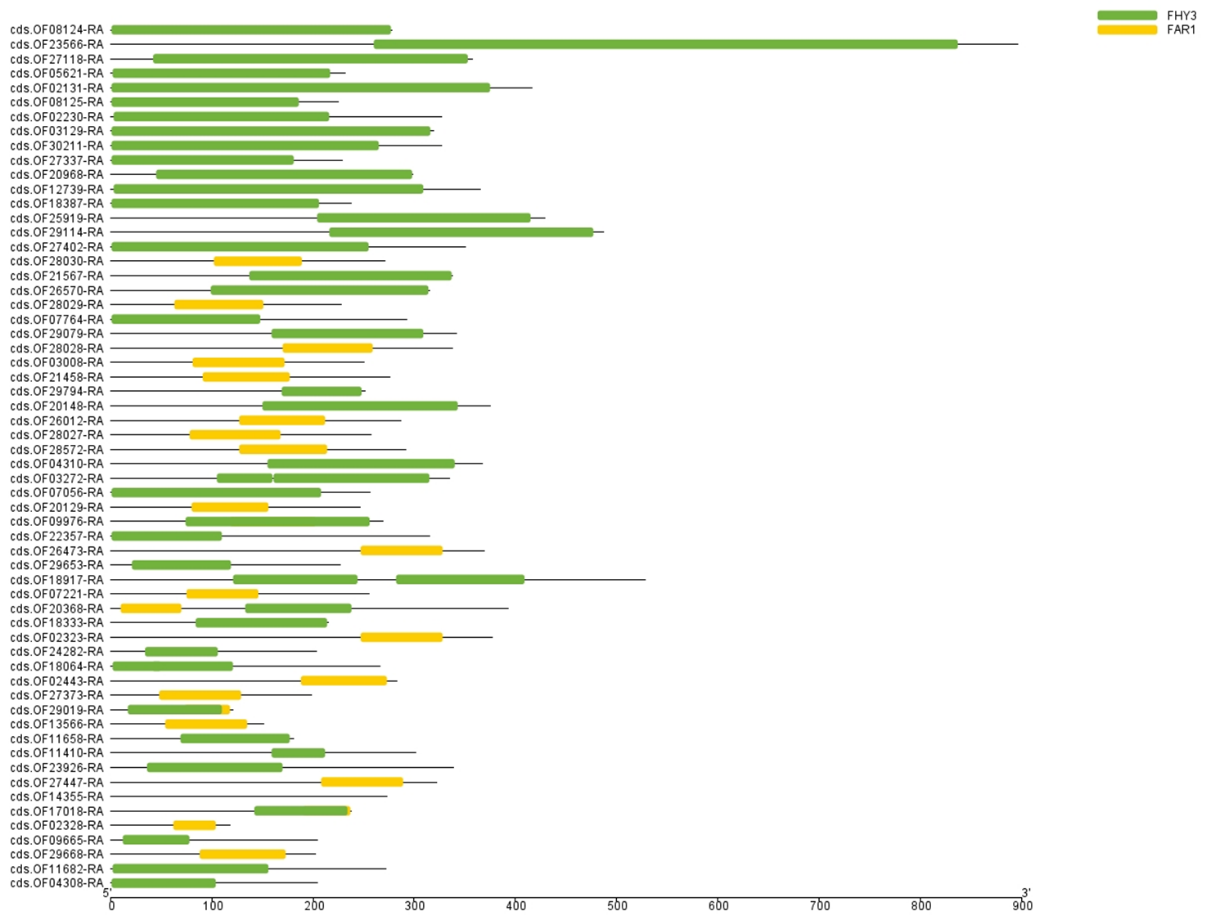


Figure S2. The conserved domain analysis of 61 family members in Walnut. Yellow bars represent FAR1 domain and green bars represent FHY3 domain.
